# Innovative strategies and nanocarrier approaches for enhancing the oral bioavailability of macromolecular therapeutics

**DOI:** 10.22038/ijbms.2025.87167.18839

**Published:** 2025

**Authors:** Pallavi Guduganahalli Manjunath, Nithin Kikkeri Ravi, Srikruthi Kunigal Sridhar, Naveen Nimbagal Raghavendra

**Affiliations:** 1 Department of Pharmaceutics, Sri Adichunchanagiri College of Pharmacy, Adichunchanagiri University, B.G.Nagara, Karnataka, 571448, India; 2 Centre of Research Management and Industrial Linkage, Adichunchanagiri University, B.G.Nagara, Karnataka, 571448, India

**Keywords:** Bioavailability, Biological products, Drug delivery systems, Gastrointestinal tract Intestinal absorption, Nanoparticles, Peptides, Receptors

## Abstract

This review focuses on identifying the primary limitations affecting the oral administration of biologics and evaluates current strategies designed to improve their bioavailability. It also incorporates a bibliometric mapping approach to assess ongoing research trends in this field. A detailed literature survey was performed alongside a bibliometric analysis using VOSviewer to illustrate keyword relationships and the evolution of research activity in oral biologic delivery. Barriers such as enzymatic degradation, restricted epithelial transport, and rapid systemic elimination hinder effective oral delivery of biologics. Innovative approaches including nanocarrier systems, enteric coatings, PEGylation, lipidation, protease inhibitors, and mucoadhesive formulations have been developed to address these issues. Advances like receptor targeted ligand systems and pH sensitive nanocarriers show promise for more efficient absorption. Emerging therapies, including GLP-1 receptor agonists and orally delivered monoclonal antibodies, are highlighted. The review also touches upon therapeutic applications beyond oncology, such as neurodegenerative diseases. Case studies on oral insulin provide valuable clinical development insights. Scaleup production and regulatory challenges are discussed, along with future directions for formulation improvement. Although oral delivery of biologics presents significant obstacles, technological advancements are steadily transforming the landscape. Future research focusing absorption mechanisms and innovative formulation strategies will be crucial for the successful development of effective oral biologic therapies.

## Introduction

A biologic is a medical product obtained from living organisms or their parts, such as blood, plasma, clotting factors, stem cells, antibodies, and vaccines, manufactured through complex biotechnological processes. They are more complex than drugs chemically synthesized and include therapeutic proteins, vaccines, gene therapies, and recombinant proteins. Biologics work through specific pharmacologic mechanisms to offer targeted treatments for any of the myriad conditions, cancers, autoimmune disorders, metabolic diseases, and so forth, which often yield fewer side effects and reduced toxicity relative to chemical drugs. Since biologics originate from biological materials, they happen to be very potent and, in general, exert their effect primarily in humans or animals. Though complex and expensive, biologics are gaining a foothold in healthcare, especially for chronic diseases (1). In addition, there is an increasing need for biosimilars, which are cheaper versions of the original biologics. The U.S. Food and Drug Administration (FDA) broadly defines biologics to include a variety of products, including vaccines, blood components, gene therapies, and somatic cells. These biologics can be made up of proteins, sugars, nucleic acids, or any combination of these substances, and may also include living entities such as cells and tissues. The pharmaceutical market’s area of biologics is an essential and expanding one because it can precisely target disease mechanisms (2).

Peptides, being short chains of amino acids, can have a wide variety of biological functions such as hormones, enzymes, or neurotransmitters. Therefore, peptides are used in medicine to their advantage as they interact with specific receptors, making them very effective for targeted therapy drugs. Some diabetes, cancer, and infections may be treated with these drugs based on peptide-based therapies. They are noticed because of the potential for being more highly specific, lessening the chances of unwanted side effects due to their capability of targeting only relevant biological pathways (3).

Proteins, which are longer and more complex than peptides, consist of long chains of amino acids folded into specific structures that enable them to perform a variety of essential functions in the body. Therapeutic proteins, such as insulin, growth factors, and clotting factors, have been pivotal in the treatment of diseases like diabetes, hemophilia, and growth disorders. As in the case of biologics, proteins can be engineered to mimic or enhance natural body processes as a method of achieving better treatments for various conditions.The advancement of medications into biosimilars and biologics in driving a transformation in the medical field, providing new hope for the treatment of complex and hard to treat diseases (4).

Biologics are best administered orally for patient compliance and convenience, but there is a major problem here: gastrointestinal obstacles. Because biotherapeutics have complicated structures and large molecules that are more susceptible to enzymatic breakdown, particularly by pepsin, which hydrolyzes peptide bonds on aromatic residues, they are often less stable in the stomach than traditional medications. In addition, pancreatic intestinal fluids, which protect IECs, affect pH shifts in different parts of the gastrointestinal tract, which can affect biologics by causing structural changes or precipitation when the molecules reach their isoelectric points. Biologics’ stability and efficacy in the intestine are further compromised by the activation of pancreatic digesting enzymes brought on by the pH rise (5).

The mucus and glycocalyx layers represent the first physical barriers the recombinant biological molecules encounter once inside the intestinal lumen. The size-exclusion filters the mucus and glycocalyx enclose, allowing large biotherapeutics not to penetrate; these will also create an unstirred layer. Biologics’ residence time in the small intestine is subsequently shortened due to the adherent layers’ ongoing turnover process. Intestinal epithelial cells beneath the mucus and glycocalyx layers also further restrict biologics permeability (6). Molecule size as well as chemical properties influence the transport of drugs across the mucosa, but biotherapeutic paracellular transport is mainly restricted to small molecules due to the tight intercellular junctions between intestinal epithelial cells. The hydrophobic interior of the phospholipid bilayer prevents biologics from dissolving because of their size, which restricts their use in carrier-mediated transport and passive diffusion. Thus, the primary method of transport across the intestinal mucosa is endocytosis or transcytosis. In particular, RME (Receptor Mediated Endocytosis) is required for effective transport through IECs(Intestinal Epithelial Cells)(7).

Apart from the physical and metabolic barriers along the GI tract, other factors such as gut microbiota or mechanical damage like osmotic stress or GI muscle peristalsis may greatly influence the bioavailability of a given drug, thus its therapeutic efficacy (8). This article aims to explore the challenges in oral biologic drug delivery, such as enzymatic degradation, limited absorption, and first-pass metabolism, while emphasizing cutting-edge strategies such as nanocarriers, enzyme inhibitors, and absorption enhancers. It touches on developments in targeted drug delivery, biologic therapies for cancer and neurodegenerative diseases, and existing constraints such as scalability and regulatory challenges. The horizon includes nano-integration, AI, and personalized medicine to improve biologic drug formulations and outcomes in patients.

### Bibliometric mapping analysis

Bibliographic mapping is a crucial method for analyzing research trends, collaborations**,** and knowledge structures in a given scientific field. This method integrates data extraction from PubMed with visualization using VOS viewer to identify to key themes, author networks, and citation patterns. Here, we outline a step by step approach to conducting bibiliographic mapping, specifically focusing on peptides, proteins, and biologics. 

First, visit PubMed (https://pubmed.ncbi.nlm.nih.gov/) and devise a full search strategy using MeSH terms and Boolean operators to filter relevant articles. The search strategy should include ((“Peptides”(MeSH) OR “Proteins”(MeSH) OR “Biologics”(MeSH)) AND (“Alzheimer Disease”(MeSH) OR “Cancer”(MeSH))) AND (bibliometric analysis OR citation analysis OR network analysis). Beyond these, add filters for certain article types (typically comments, clinical trials, or meta-analyses); filter by date range (for example, the last decade); and leave that option for language (English). When you’ve finished searching for results, go to Send to → File and get either XML or RIS to download citation data (9)**.**

After downloading the PubMed data file, open VOS viewer (https://www.vosviewer.com/) and starting a new bibliometric analysis. Select “Create a map based on bibliographic data”, then choose “Read data from bibliographic database files” to upload the downloaded XML or RIS file. At this stage, VOS viewer allows users to select different types of analyses, such as co-authorship analysis (to identify collaborations among authors or institutions), co-occurrence of keywords (to reveal frequently used terms and research themes), citation analysis (to show the most cited papers), and co-citation or bibliographic coupling (to map relationships between articles based on shared citations). Once the data has been processed, the threshold settings should be changed, such that at least some specific minimum number of occurrences for terms or citations will be included in the analysis-potentially as much as, for example, authors who have been cited at least five times (10). Alter node and link strength according to the focus of the connections between topics or researchers. VOS viewer generates a network visualization showing the linkages between authors, keywords, or institutions, overlay visualizations for research trends over time (older vs. newer), and density visualizations, indicating research hotspots-high publication volume areas. By analyzing these maps, researchers can identify key authors and leading research institutions, recognize emerging research themes in peptides, proteins, biologics, Alzheimer’s disease, and cancer, and detect gaps in the existing literature and potential areas for further study (11)**.**

**Figure 1:**
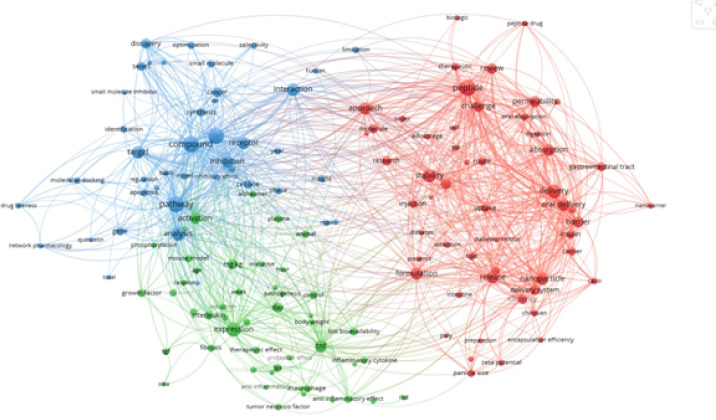
Bibliometric mapping analysis showcasing research trends, keyword co-occurrences, and collaborative networks in the field

The bibliometric analysis can subsequently be exported as PNG or CSV files to complete the reporting. VOS viewer also provides statistics to assist in understanding trends. These data can be used in systematic reviews and meta-analyses, in research proposals and grant applications, or for identifying influential studies and research clusters (Figure 1). Employing such a structured approach enables researchers to effectively visualize and interpret the scientific landscape as regards studies pertaining to peptides, proteins, biologics, Alzheimer’s disease, and cancer alike vis-a-vis data-oriented decision-making in research planning (12)**.**

A bibliometric analysis was performed using VOSviewer on data sourced from PUBMED, spanning publications from the past ten years. The study examined keyword relationship, author collaborations and publication patterns. The resulting visualizations, shown in Figure 1, highlight significant research areas and connections, strengthening the study’s validity and clarity (13).

### Challenges in oral delivery

*Biologics*

Biologics, including monoclonal antibodies, gene therapy, vaccines, and recombinant proteins, have transformed modern medicine by providing targeted and highly effective treatments for various diseases. However, their oral delivery remains a significant challenge due to multiple physiological and biochemical barriers in the gastrointestinal (GI) tract (1). Biologics are high-molecular-weight hydrophilic substances with complex structures, making their absorption through the intestinal epithelium highly inefficient. Unlike smaller molecules that diffuse easily across cell membranes, biologics face significant barriers due to mucosal layers, enzymatic degradation, and the absence of active transporters, leading to poor bioavailability. Additionally, their structural instability in the acidic stomach environment (pH ~1-3) results in denaturation and enzymatic breakdown by gastric and pancreatic proteases, such as pepsin, trypsin, chymotrypsin, and carboxypeptidases, further limiting their systemic absorption (14). Immunogenicity is another challenge, as biologics derived from recombinant DNA technology or non-human sources can trigger immune responses, leading to the production of anti-drug antibodies (ADAs) that neutralize therapeutic effects and potentially cause adverse reactions. Besides, the short half-lives of biologics are due to rapid degradation by proteolytic enzymes in the gastrointestinal tract, liver, and kidney, leading to rapid clearance from circulation. Consequently, biologics are often administered more frequently or based upon advanced drug delivery techniques for stability enhancement and extended half-life, which include nanoparticle carriers, lipid-based carriers, or polymeric systems (15).

***Proteins***

Proteins and peptides are confronted with numerous difficulties in oral administration because they are degraded by enzymes, poorly permeable, and have brief circulation periods. The macromolecules are large and hydrophilic in nature, which complicates their ability to penetrate through the intestinal epithelium. In contrast to small-molecular-weight drugs that pass through cell membranes by diffusion, proteins and peptides are incapable of passing through tight junctions of the intestinal lining and have no effective transporter systems within the gut that further limit their systemic absorption. Moreover, their hydrophilicity and size bar passive diffusion across the intestinal membrane, and lacking specific transporters further restricts their absorption. In systemic circulation, proteins and peptides typically have low half-lives due to extensive kidney and liver clearance. Their large molecular weight exposes them to renal filtration, and the reticuloendothelial system (RES) targets and degrades them. To ensure therapeutic levels, they need to be administered frequently or in sustained-release forms to increase their stability and extend their circulation time(16).

***Peptides***

**Figure 2:**
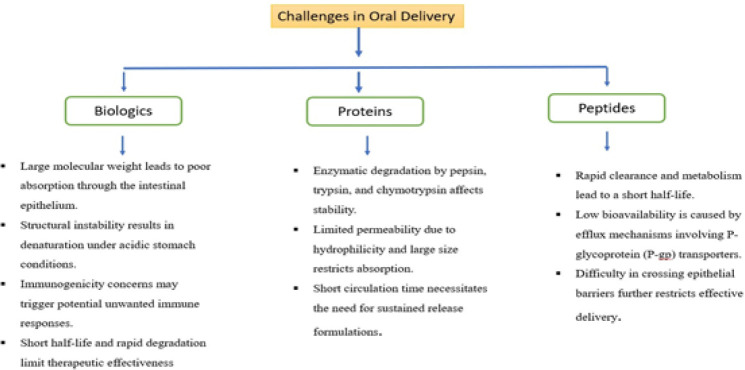
Challenges in oral delivery of biologics, proteins, and peptides, including poor absorption, enzymatic degradation, structural instability, immunogenicity, and short half-life

Peptides, encompassing mini-therapeutics and hormones like GLP-1 agonists, are highly promising for the treatment of chronic, endocrine, and metabolic diseases but are challenging to deliver orally because of numerous physiological and biochemical barriers that impede absorption, stability, and bioavailability. The biggest drawback is their quick enzymatic digestion and clearance with a resultant short half-life. Proteases and peptidases like dipeptidyl peptidase-IV (DPP-IV), aminopeptidases, and carboxypeptidases in the gut and blood degrade peptides prior to systemic circulation. Furthermore, renal filtration also clears peptides quickly, and hepatic metabolism further alters and inactivates them, making frequent dosing or other formulations like sustained-release systems necessary to ensure therapeutic levels (Figure 2). In addition, peptides are hampered by poor oral bioavailability owing to efflux by P-glycoprotein (P-gp) transporters, which actively efflux them from enterocytes into the gut lumen, excluding absorption. Those that reach systemic circulation are subjected to extensive first-pass metabolism by the liver and intestine, reducing their efficacy even further. The other major challenge is their inability to cross the intestinal epithelium because of their large molecular size and hydrophilicity, which hinder passive diffusion across lipophilic cell membranes. Tight junctions between intestinal epithelial cells restrict paracellular transport, and the lack of specific transport proteins further limits their uptake. These challenges collectively reduce the oral bioavailability of peptides, necessitating innovative drug delivery strategies to enhance their absorption and therapeutic efficacy (17).

### trategies to enhance oral bioavailability

Strategies for biologics

***Nanocarrier-based delivery***

In biomedicine, liposomes have drawn a lot of interest, especially as anticancer drug delivery vehicles. They offer numerous advantages, including enhanced drug distribution, protection from environmental degradation, improved product efficacy, and reduced systemic toxicity. When compared to free pharmaceuticals in solution, medications encapsulated in liposomes show different pharmacokinetics, with PEGylation prolonging circulation duration. Liposomes, also referred to as unilamellar or multilamellar vesicles, respectively, are spherical vesicles containing an aqueous interior covered by lipid bilayers that may consist of one or more layers. Their biological utility is supplemented by their ability to encapsulate water- and lipid-soluble medicines. As indicated by doxorubicin-loaded PEGylated liposomes, which increase tumor drug concentration and decrease systemic toxicity, drug-loaded liposomes maximize biodistribution and pharmacokinetics. For targeted drug delivery, liposomes can also be attached to ligands or antibodies. Another category of drug carriers, polymeric nanoparticles (PNPs), has gained attention due to their controlled release properties, enhanced drug payload, and stability. PNPs are colloidal particles between 10 and 1,000 nm in size and made of biodegradable polymers. They can be categorized as either nanospheres, which confine medicines within a polymer matrix, or nanocapsules, which contain drugs in an inner liquid core surrounded by a polymer shell. Solvent evaporation, nanoprecipitation, dialysis, and polymerization methods are some of the various means through which PNP can be synthesized. Synthetic biodegradable polymers such as polylactic acid (PLA), polyglycolic acid (PGA), PLGA, PEG, and polycaprolactone (PCL) and natural polymers such as chitosan, alginate, gelatin, and heparin are commonly used in PNPs. PNPs exhibit better stability, higher drug loading, controlled physicochemical properties, longer circulation times, and better drug release compared to other nanocarriers such as liposomes and polymeric micelles. These characteristics guarantee efficient and long-lasting therapeutic delivery, which makes PNPs extremely appealing for the treatment of cancer (18).

***Enteric coating*** ***&*** ***encapsulation***

Important pharmaceutical processes that shield medications from stomach breakdown and allow for targeted intestine release are enteric coating and encapsulation. Enteric coatings employ pH-sensitive polymers, such as Hydroxypropyl Methylcellulose Phthalate (HPMCP), Cellulose Acetate Phthalate (CAP), and Eudragit® (methacrylate copolymer), which dissolve at intestinal pH (5.5–6.5) but remain insoluble in acidic gastric conditions (pH<5). These coatings enable for site-specific medication release, improve bioavailability, and avoid gastrointestinal discomfort. For colon-targeted delivery, eudragit-based coatings are frequently utilized; Li *et al.* showed that eudragit S100 is effective for 5-fluorouracil and leucovorin, guaranteeing site-specific release (19). Similarly, Singh *et al.* used Eudragit RL 100 to increase the oral bioavailability of atazanavir. CAP is used in protein, peptide, and vaccine nanoencapsulation as well as protecting acid-sensitive medications (20). Through diffusion and erosion mechanisms, HPMC controls drug release; Katzhendler *et al.* demonstrated that its molecular weight affects naproxen release, enhancing drug solubility at high pH. To improve stability, regulate medication release, and boost therapeutic efficacy, encapsulation entails encasing active substances in carrier materials such as hydrogels, liposomes, or nanoparticles (21). These tactics lessen systemic toxicity, increase circulation time, and maximize medication distribution. Enteric coating and encapsulation greatly enhance pharmaceutical formulations by preventing premature drug breakdown and guaranteeing accurate release, particularly for medications that are sensitive to stomach conditions. These technologies are essential to contemporary medication delivery systems because they improve treatment results and patient compliance (22).

***Antibody delivery systems***

Therapeutic antibodies face significant challenges for oral administration because of their limited absorption and degradation within the gastrointestinal tract. Through the facilitation of transcytosis through the intestinal epithelium, the neonatal Fc receptor (FcRn)-targeted method offers a promising solution. Insulin G (IgG) is recycled and transported past epithelial barriers by FcRn, which is endogenously produced in the gut. The development of monoclonal antibodies (mAbs) or antibody fragments that have a high affinity for FcRn at the intestinal acidic pH (~6.0) can result in efficient absorption. In the circulation, these antibodies dissolve at neutral pH (~7.4), reproducing maternal IgG transport in newborns (23). Scientists have created nanocarriers such as polymeric nanoparticles and liposomes targeted to FcRn to increase stability and uptake. Fc-fusion proteins, enteric-coated formulations, and polymer-based drug delivery systems are some recent advancements that provide intestine release targeting and protect antibodies from enzymatic degradation. Moreover, bioavailability has been improved by the inclusion of FcRn-targeting ligands, permeability enhancers, and mucoadhesive polymers. These advancements have enormous potential for oral antibody delivery to treat long-term illnesses such autoimmune diseases and inflammatory gastrointestinal disorders. As research progresses, FcRn-targeted strategies may transform the field of antibody therapies by providing a more patient-friendly alternative to injections (24). 

***Smart hydrogels*** ***&*** ***injectable tablets***

Reversible, intensity-scalable, reproducible, and controllable phase volume transitions are made possible by the nonlinear feedback that smart hydrogels exhibit in response to tiny external stimuli, which causes abrupt changes in their physical characteristics and macroscopic structure. When the trigger is withdrawn, smart hydrogels regain their previous structure through modifications to their physical state, hydrophilicity, conductivity, shape, solubility, and solvent interaction. Because they prevent drug buildup in non-target tissues, their application in drug delivery systems offers significant advantages such less frequent dosing, maintained therapeutic drug levels with a single dose, and fewer side effects. Additionally, smart hydrogels are easy to make and are great options for drug-embedded long-release systems. With a focus on a few chosen triggers for clever drug delivery applications, this study aims to highlight current breakthroughs and significant advancements in stimuli-responsive hydrogels during the previous five years rather than providing a thorough overview of the broad area of hydrogels (25).

### Strategies for proteins

***PEGylation*** ***&*** ***lipidation***

Two crucial techniques for improving the stability and extending the half-life of therapeutic proteins are lipidation and PEGylation. By binding lipid moieties to proteins, lipidation promotes robust albumin binding and inhibits enzymatic breakdown and quick renal clearance. As exemplified by lipidated glucagon-like peptide-1 (GLP-1) analogues liraglutide and semaglutide, whose half-lives are 13 and 168 hours, respectively, this approach significantly extends circulation time. In the same vein, due to their prolonged 40-hour half-life, lipidated human growth hormone mimetics allow for weekly dosing. Lipidated proteins are also shielded from degradation by albumin binding via neonatal Fc receptors (FcRn). Conversely, PEGylation involves covalent attachment of polyethylene glycol (PEG) chains to proteins, which inhibits enzymatic degradation, reduces immunogenicity, and reduces renal clearance. Such drugs as PEG-asparaginase (Oncaspar®) and PEG adenosine deaminase (Adagen®) exhibit improved pharmacokinetics due to this method, which leads to increased circulation times and reduced dosing. Site-specific modifications can now be achieved due to advancements in PEGylation chemistry that preserve protein function while minimizing the formation of unwanted isomers. The effectiveness of PEGylation is further evidenced by medications such as PEGylated interferons for hepatitis C and Pegfilgrastim® for neutropenia. Both methods are essential to contemporary biopharmaceuticals since they guarantee longer-lasting medication action, improved stability, and improved patient adherence (26).

*Enzyme inhibitors for proteolytic degradation*

Protease inhibitors can be used together to reduce the enzymatic barrier and stop proteins and peptides from breaking down. The two-pulse delivery method schematically: Intermediary enzyme inhibitor/absorption enhancer (c), inner HPMC coating (b), protein-containing core (a), and outside HPMC coating (d). Permittedly reproduced from reference (8). In the GI tract, making intestinal absorption easier. The stability of protein and peptide medications is assessed mostly by the molecule’s structure and enzyme specificity. Amastatin, bestatin, boroleucine, puromycin, and aprotinin (trypsin/chymotrypsin inhibitor) are examples of inhibitors that have been used extensively. Among the relatively harmless naturally occurring inhibitors, aprotinin has found widespread application in the oral administration of peptides. Trypsin and chymotrypsin inhibitors increased the oral bioavailability of insulin when given together. The impact of enzyme inhibitors, including sodium glycocholate, camostat mesilate, bacitracin, soybean trypsin inhibitor, and aprotinin, on the intestinal metabolism of insulin in rats was investigated (27). Bacitracin, sodium glycocholate, and camostat mesilate were found to be more effective in increasing the large intestine’s physiological availability of insulin. 

With D-conformation of alanine and leucine amino acids, the oral bioavailability of the pentapeptide enkephalin YAGFL (Tyr-Ala-Gly-Phe-Leu) increased significantly (22-fold) when peptidase inhibitor amastatin was present. Since most natural inhibitors are prone to enzymatic breakdown in the stomach, they must be co-administered in extremely high dosages. It may be necessary to encapsulate proteins and peptides in nano-drug delivery devices since even high concentrations of these inhibitors may not be sufficient to lower protease activity (28). The long-term, chronic use of these inhibitors, however, may lead to significant toxicity. Additionally, it might impact the absorption of other proteins that would typically be broken down. These inhibitors have a significant disadvantage in that their nonsite specific activity will significantly change the intestinal membrane’s and GI tract’s metabolic pattern, mainly because dietary proteins are not as well broken down (29)(Table 1).

***Mucoadhesive*** ***&*** ***intestinal targeting systems***

**Figure 3:**
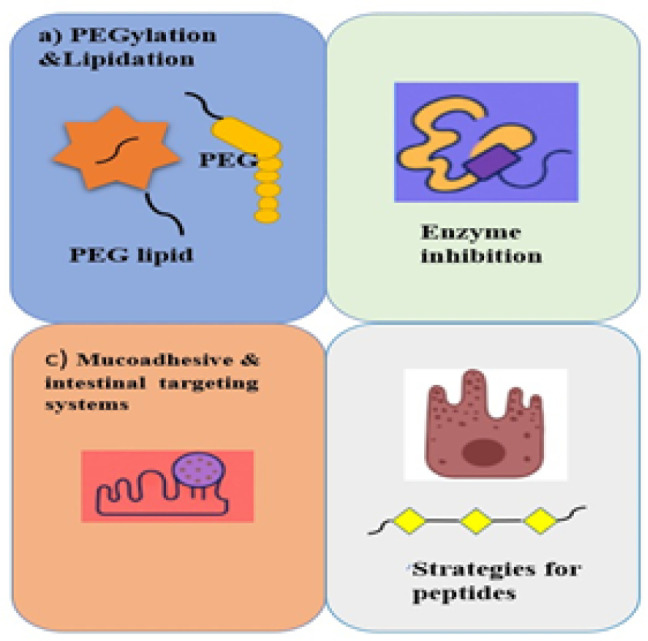
(a) PEGylation & Lipidation Enhancing stability and half-life by modifying proteins with PEG chains or lipid moieties. (b) Enzyme Inhibitors Protecting peptides from proteolytic degradation using inhibitors like aprotinin and amastatin. (c) Mucoadhesive & Intestinal Targeting Systems Increasing absorption via mucoadhesive polymers and targeted drug delivery. (d) Peptide Stabilization Strategies Improving bioavailability using chemical modifications like cyclization, N-methylation, and lipidation

Intestinal and mucoadhesive targeting systems are innovative techniques designed to improve drug efficacy and bioavailability by retaining therapeutic molecules at the site of absorption. Drugs can adhere to the GI tract for an extended period of time thanks to mucoadhesive systems, which use polymers that interact with the mucosal layer to prolong absorption. Enzyme-activated release systems and pH-sensitive coatings are two examples of intestinal targeting techniques that offer site-specific medication delivery, minimizing degradation and boosting therapeutic effects (Figure 3). These tactics are particularly helpful for proteins and peptides, which are otherwise subject to poor GI tract absorption and enzymatic breakdown. Mucoadhesive and intestine targeting devices significantly improve oral medication administration by increasing residence time and targeted release (30). 

### Strategies for peptides

Due to issues like low bioavailability, quick enzymatic breakdown, and restricted permeability, peptide-based treatments require methods to improve their stability, absorption, and effectiveness. By adding alterations such as N-methylation, D-amino acid replacement, cyclization, and β-amino acid insertion, peptididomimetics—synthetic analogs of natural peptides—improve metabolic stability, prolong peptide half-life, and avoid protease recognition. As demonstrated by medications like linaclotide and bortezomib, these backbone changes also improve receptor binding affinity and selectivity (Table 1). Because fatty acid conjugation increases peptide lipophilicity and facilitates albumin binding, which prolongs circulation time, it improves oral bioavailability and membrane permeability. Fatty acid attachment enhances stability and absorption through the transcellular pathway in oral semaglutide, which is a prime example (31). Peptide transport is further optimized by lipidation methods like myristoylation and palmitoylation. Furthermore, by creating fine emulsions during ingestion, Self-Emulsifying Drug Delivery Systems (SEDDS) improve intestinal absorption and solubility by facilitating drug dissolution and absorption across intestinal membranes. Poorly soluble peptides are particularly well served by this method, which also enhances their bioavailability and therapeutic action. Together, these strategies assist in overcoming the inherent limitations of peptide-based medicines, ensuring better stability, controlled release, and greater efficacy (32).

### Advances in oral biologics and peptides

*Role of nanocarriers in overcoming gastrointestinal barrier*

Nanocarriers play a crucial role in biologics with regard to augmenting drug delivery, stability, and therapeutic action. Nanocarriers enable targeted drug delivery through ligand-receptor binding, increasing site-specific activity at the cost of off-target interactions. Through protection of biologics from enzymatic degradation and excess clearance, nanocarriers enhance bioavailability and circulation half-life. They also provide controlled and sustained delivery, obviating the necessity of frequent dosing and minimizing toxicity. Additionally, nanocarriers reduce immunogenicity and toxicity by shielding biologics from immune recognition (33). Their capacity for crossing biological barriers, including the blood-brain barrier, provides intracellular delivery of drugs to treat complicated diseases. Moreover, nanocarriers facilitate simultaneous co-delivery of multiple drugs, enhancing outcomes in cancer therapy, gene therapy, and vaccine delivery. Theranostics utilizes them as contrast agents to image and real-time monitor drug distribution and therapeutic response (34). Oral administration of biologic drugs such as proteins, peptides, and nucleic acids is desirable as it is less invasive and enhances compliance in patients. The GI tract, however, poses huge hurdles to efficient transportation of the big molecules, ranging from enzymatic degradation to low pH, limited permeability, and physical impediments. 

**Figure 4:**
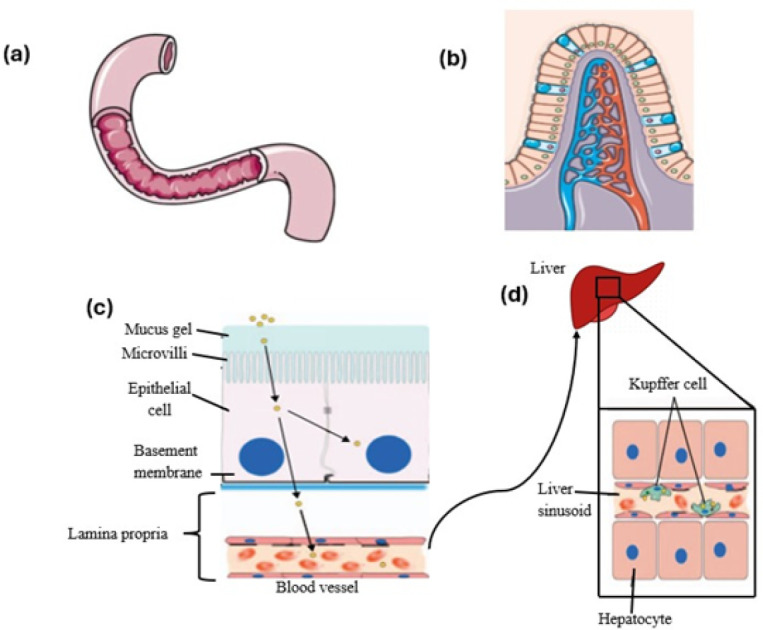
(a) In the small intestine, pH-dependent enteric coatings dissolve, and peptide-based macromolecules need protection from pancreatic juice using citric acid or protease inhibitors. (b) which consists primarily of enterocytes and goblet cells. All BCS Class III molecules require assistance with permeation to pass across the epithelial surface. (c) Absorption enhancers increase epithelial permeability, allowing macromolecules to enter capillaries and the hepatic portal vein, where the first-pass effect may reduce absorption. (d) If a macromolecule is encapsulated in a nano- or micro-particulate, further barriers to absorption are the basement membrane, the endothelium of capillaries, the hepatic portal vein and the Kupffer cells that line sinusoids

A viable means to overcome such obstacles and boost the oral bioavailability of biologics is with the use of nanocarriers. One of the largest challenges is the stomach’s acidic environment, where biologics are destroyed by digestive enzymes like pepsin and the acidic pH. Nanocarriers, including pH-sensitive nanoparticles and liposomes, enhance stability and bioavailability by protecting biologics in a shell and delivering their payload in the relatively more neutral small intestine environment (35). Another obstacle is the mucosal barrier, which is made up of the glycocalyx and mucus layers and limits the access of large molecules to epithelial cells. Nanocarriers with hydrophilic coatings like polyethylene glycol (PEG) have mucus-penetrating properties that increase diffusion and allow transit through these layers (Figure 4). Once passed through the mucosal barrier, biologics face tight junctions in the intestinal epithelium, which restrict paracellular transport. Nanocarriers are capable of facilitating endocytosis and transcytosis-mediated uptake and can be functionalized for targeted delivery. Moreover, enzyme-resistant parts of nanocarriers prevent biologics from degradation, enhancing their bioavailability and absorption even further (36).

*Advances in targeted delivery systems*

The goal of targeted medication delivery systems is to maximize medicinal efficacy and minimize negative effects by accurately delivering therapeutic molecules to particular bodily locations. In oral medication administration, two sophisticated approaches have been developed to improve bioavailability, especially for medicines that have poor absorption or solubility: pH-sensitive and stimuli-responsive nanocarriers and ligand-based targeting for intestinal receptors (37).

*Ligand-based targeting for intestinal receptors*

Ligand-based targeting utilizes specific chemical interactions between ligands (e.g., peptides, antibodies, and small molecules) and intestinal cell surface receptors. This approach ensures that the drug is delivered effectively to its target site via the digestive epithelium. Receptor-mediated endocytosis, where ligands bind to intestinal cell receptors to induce the drug-receptor complex to be taken inside, is an important mechanism in ligand-based targeting. The drug can be directly delivered to the target tissues or released into systemic circulation after entry into the cells(38). For increased drug absorption, receptors like the transferrin receptor, vitamin B12 receptors (Cubilin), and follicle-stimulating hormone receptor (FSHR) are targeted. The employment of cell-penetrating peptides (CPPs) or absorptive-mediated peptides (AMPs) in peptide-based targeting enhances medication uptake further. Additionally, the bioavailability of drugs that are poorly absorbed, such as hydrophilic compounds or proteins, can be improved by ligand conjugation with nanoparticle drug delivery systems (31). For example, folic acid- or transferrin-functionalized nanoparticles are able to target specific cells that bear these receptors for enhanced drug absorption. Intestinal disease targeted therapy is facilitated by the primary benefits of ligand-based targeting, which are increased absorption of drugs with poor absorption, reduced systemic toxicity, and increased specificity (39).

*pH-sensitive and stimuli-responsive nanocarriers*

Oral medication delivery using pH-sensitive and stimuli-responsive nanocarriers is another cutting-edge technique. These nanostructures are engineered to release their therapeutic payload in reaction to changes in temperature, pH, or certain enzymes in the environment. Because they must go through the more alkaline small intestine and the acidic stomach, pH-sensitive carriers are especially helpful for oral administration. When exposed to the alkaline pH of the colon or the acidic circumstances of the stomach, these carriers release the medication, although they remain stable in neutral pH environments (40). Polymeric micelles, liposomes, and hydrogels are frequently utilized materials. Polymers that expand and release the medication at certain pH levels include poly(methacrylic acid) and Eudragit L100-55. Stimuli-responsive nanocarriers that respond to enzymes or temperature are also becoming more and more popular. Drugs can be released by thermo-responsive polymers at body temperature or by enzyme-responsive nanocarriers when certain enzymes are present in the gut or tumors. These technologies improve bioavailability and reduce negative effects by enabling regulated medication release at the target site (41).

*GLP-1 receptor agonists (Semaglutide)*

An intestinal hormone belonging to the incretin family, GLP-1 increases insulin production, inhibits glucagon release, postpones stomach emptying, and lowers insulin resistance (42). GLP-1 is a neurotransmitter in the central nervous system that communicates feelings of fullness through the brainstem-hypothalamus. Peripherally, GLP-1 lowers energy intake and influences all aspects of appetite regulation, including a decrease in hunger and future food intake as well as an increase in satiety and fullness (43). These effects work together to encourage physiological satiety. Due to the extremely low bioavailability of peptide-based medications, such as GLP-1 RA, when taken orally, this effective treatment option for type 2 diabetes was only available in parenteral formulations (44). T2D can be treated with semaglutide, a GLP-1 RA, administered subcutaneously once per week (30, 31). This injectable compound is a powerful, long acting GLP-1 analog that shares 94% sequence similarity within native human GLP-1.The extended pharmacokinetics of this drug are due to three important structural differences: the attachment of a linker and C18 di-acid chain at position 26 that provides strong binding to albumin; the substitution of Lys with Arg at position 34 that prevents C18 fatty acid-binding at the wrong site; and the substitution of Ala with Aib at position 8 that increases enzymatic (DPP4) stability (45). 

In individuals with type 2 diabetes, subcutaneous semaglutide was compared to a placebo or other active standard-of-care drugs in the SUSTAIN phase 3 clinical research program. These studies demonstrated that the subcutaneous semaglutide group saw a higher decrease in HbA1c and body weight loss rates, as well as a well-defined safety profile. But T2D needs to be treated for a long time, and patients generally prefer oral formulations, which may help with medication adherence (46). According to the PIONEER program, a series of phase III clinical trials demonstrating the effectiveness and safety of oral semaglutide in comparison with placebo or other active standard-of-care medications, the Food and Drug Administration (FDA) approved the oral formulation of semaglutide on September 20, 2019, and the Brazilian Health Regulatory Agency (ANVISA) approved it on October 20, 2020, to treat type 2 diabetes (33, 34). The oral formulation’s bioavailability was guaranteed by the coformulation of semaglutide with sodium N-(8-(2-hydroxybenzoyl) amino) caprylate (SNAC), a transcellular permeation enhancer (44). SNAC has a primarily transcellular transit mechanism across the stomach epithelium in conjunction with semaglutide; absorption takes place 60–140 minutes after a pill containing 300 mg of SNAC and 10 mg of semaglutide is consumed in humans and dogs (19). Since SNAC raises the local stomach pH, it most likely also reduces semaglutide enzymatic digestion. Phase I clinical trials demonstrated that oral semaglutide pharmacokinetics are unaffected by concurrent use of omeprazole, contraceptive pills, or other medications frequently used for type 2 diabetes, as well as renal failure of any severity (47). Patients should take oral semaglutide in the morning when fasting and with up to half a glass of water (around 120 mL), at least 30 minutes before consuming any food or other drugs, to guarantee bioavailability. To reduce the risk of gastrointestinal side effects, patients should progressively increase their oral semaglutide dosage, starting at 3 mg once day and working their way up to 7 mg and, if required, 14 mg once daily after 30 days (48).

*Oral monoclonal antibodies in clinical trials*

Monoclonal antibodies (mAbs) have changed the treatment of autoimmune disorders, cancers, and infectious diseases. Conventionally administered by IV or SC routes due to their large size and their susceptibility to enzymatic degradation in the GI tract, recent advancements in oral biologics have opened the way to develop oral delivery of mAbs currently in clinical trials (49). The oral route for mAbs holds promise; however, they meet with hurdles in the gastrointestinal environment; these include degradation by gastric and intestinal enzymes (such as proteases and peptidases), poor absorption due to their large molecular size across intestinal epithelium, and bioavailability, which necessitates very high doses to elicit a therapeutic effect. Hence, novel delivery technologies-including nanoparticles and liposomes-were developed to encapsulate and deliver mAbs to negate exposure to electrolytic enzymatic degradation (50). Examples of such models are PEGylated nanoparticles and lipid delivery systems. Receptor-mediated transport systems, which bypass the intestinal barrier using intestinal Fc receptor (FcRn) for transporting intact mAbs, seem to prove effective in FcRn-directed oral antibody therapies; others involve using permeation enhancers and enzyme inhibitors such as SNAC and bile salt derivatives for absorption enhancement via decreased enzymatic degradation (51).

Development of micro-needle capsules and auto-injecting pills, such as RaniPill®, has promoted the direct delivery of mAb to the intestinal wall. Clinical trials are currently ongoing for several oral mAbs being evaluated, including oral adalimumab in inflammatory diseases, oral vedolizumab for ulcerative colitis and Crohn’s disease, and oral anti-IL-23/IL-17 mAbs for psoriasis and inflammatory bowel disease. Early research is being conducted for the use of monoclonal antibodies orally for the treatment of COVID-19 by targeting the spike protein of SARS-CoV-2 (51). A considerable prospect awaits oral mAbs in the future pertaining to cancer therapy, where immune checkpoint inhibitors could be developed in oral form, like PD-1 and CTLA-4 inhibitors. Oral mAbs could prove effective against metabolic diseases targeting inflammatory pathways in obesity and diabetes. There is also an ongoing exploration of combination therapies with oral mAbs with co-administered small molecule drugs to increase efficacy. The successful commercialization of oral monoclonal antibodies would change biologic therapies for the better by improving accessibility and patient compliance as well as by broadening the treatment possibilities. Despite challenges, ongoing advances in drug delivery and formulation technologies are paving the way for oral mAbs to make it to the clinic, rightly standing as a giant leap in biologic therapeutics (52).

### Therapeutic applications of oral biologics: Advances in neurodegenerative disorders and cancer

**Figure 5:**
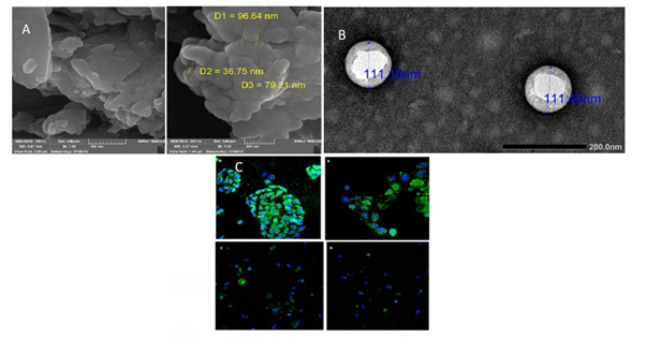
(a) FE-SEM of optimum solid nanoemulsion (SNE-2)(Reproduced from (55)). (b) Transmission electron microscopy (TEM) photograph of the optimized AST–NLC formulation (40,000X magnification)(Reproduced from (56)). (c) Confocal microscopy shows higher uptake of targeted NPs in MCF-7 cells. (A, B) compared to non-targeted ones, while no significant difference is observed in SKOV-3 cells (C, D)(Reproduced from (57))

Oral biologics have emerged as a revolutionary modality in the treatment of many diseases such as neurodegenerative diseases and cancer. Most biologic drugs, such as monoclonal antibodies and peptides, are used through intravenous (IV) or subcutaneous (SC) injection, considering all these factors: enzymatic degradation resistance and poor absorption by the GI tract. Advancements in technology for drug delivery have however opened up possibilities for oral formulations, which promise greater convenience to the patient, better compliance, and long-term management of diseases. The types of neurodegenerative diseases include Alzheimer’s disease (AD), Parkinson’s disease (PD), and amyotrophic lateral sclerosis (ALS). These diseases represent quite a treatment challenge due to the effective drug delivery required by the blood-brain barrier (BBB)(53). Current innovations in oral biologics include nanoparticles, liposomal carriers, and receptor-mediated transport systems aimed at enhancing drug stability and hence facilitating penetration of the BBB with recent techniques. GLP-1 receptor agonists, including semaglutide, are examples of drugs that have been shown to decrease neuroinflammation and oxidative stress, and exhibit neuroprotective effects in AD and PD. Some oral monoclonal antibodies (mAbs) that target both beta amyloids (Aβ) and tau proteins in AD are being studied to slow disease progression (Figure 5). 

Oral siRNA and antisense oligonucleotides are RNA-based therapies that are envisaged to modulate gene expression related to disease in neurodegenerative disorders. As far as anticancer therapy is concerned, they develop oral biologics to improve drug bioavailability and bio-targeting with a minimum adverse effect. Oral versions of checkpoint inhibitors, including PD-1/PD-L1 and CTLA-4 inhibitors, are being studied for easy access and minimizing hospital visits. Moreover, they are developing oral oncolytic peptides that are specified against cancer cell receptors such as integrins to inhibit tumor growth and metastasis (54). Also, oral biologic vaccines are under development for personalized cancer therapy, utilizing immune-stimulating peptides. The successful development of oral biologics for the treatment of neurodegenerative and cancer diseases would be a big leap in precision medicine, bringing patients back into hope with chronic and possibly life-threatening diseases. Much work remains to be done, but research continues on formulation technologies and drug delivery, putting us on the path to a future where oral biologics will continue to be the central focus of disease management. Neurodegenerative diseases and cancer are among the most challenging conditions to treat due to their complex pathology, limited drug penetration, and high unmet medical need. Advances in biologics and peptide therapies specifically target these diseases, offering innovative treatment strategies to overcome traditional therapeutic limitations.

### Case studies

*Oral insulin developments (Oramed, Novo Nordisk)*

On account of the destruction by enzymes, ineffective intestinal absorption, and rapid clearance, the oral insulin development has been a prominent challenge. Oramed Pharmaceuticals has developed an oral insulin capsule (ORMD-0801) using protective coating technologies and absorption enhancers to improve bioavailability. Novo Nordisk has ventured into oral insulin formulations as well, applying its experience with oral semaglutide to develop carrier technologies that stabilize the drug and enhance its uptake through the intestinal tract. These breakthroughs are intended to revolutionize the treatment of diabetes by providing a non-invasive alternative to injecting insulin, thus improving patient adherence and quality of life.

*Case studies and recent advances*

“The Effect of Anti-Tumor Necrosis Factor Agents on Surgical Stress Response in Inflammatory Bowel Disease Patients Undergoing Abdominal Surgery” (NCT01974869), funded by El-Hussuna, Alaa, M.D., sought to assess the effect of anti-Tumor Necrosis Factor-alpha (TNF-α) agents on the surgical stress response in patients with Crohn’s disease, ulcerative colitis, and indeterminate colitis who are having elective abdominal surgery (laparoscopic or open).This future, unblinded, non-interventional cohort study, Performed at a single institution, the trial included 47 patients and evaluated both immunological and endocrinological parameters to address if TNF-α inhibitors have an effect on surgical stress response and postoperative outcomes. Preoperative blood samples, during anesthesia induction, and at 6, 24, and 48 hours postoperatively were taken to assess inflammatory cytokines (TNF-α, IL-1, IL-6, IL-10), C-reactive protein, and white blood cell count, as well as endocrinological markers like plasma cortisol, growth hormone, adrenocorticotropic hormone, epinephrine, and norepinephrine. Patient factors were assessed through demographic and clinical information, as well as through validated scoring systems such as the Charlson Morbidity Index for co-morbidities, the Nutritional Risk Score (NRS) for nutritional status, and the Harvey-Bradshaw Index (HBI) for Crohn’s disease activity. Surgical factors, such as duration and amount of blood transfusion, were also noted. The primary outcome of the study was the inflammatory cytokine change, with secondary outcomes being 30-day postoperative complications. Using a power calculation, 17 patients per group were needed for statistical viability. The study preserved biospecimens with DNA and enrolled all eligible patients, without exclusions by age or sex. The results were intended to help understand the immunologic impact of biologics during surgical stress and how they may influence postoperative recovery. 

The “Collection of Biological Specimens and Associated Health Information for Secondary Research in Future Studies” (NCT06369441) research, funded by Ovation.io, Inc., is a prospective, case-only observational study with the aim to collect whole blood samples from adult cancer patients (≥18 years old) with documented diagnoses of solid tumors or hematologic malignancies. The main purpose is to build a biorepository that will enable future investigations of cancer diagnosis, therapy, and the genes controlling disease and health outcomes. The research holds biospecimens with DNA up to 10 years, facilitating secondary research on oncology and precision medicine. Participation is restricted to patients undergoing care at participating sites of care, and those individuals who cannot give informed consent or are incarcerated at the sample collection phase are not included. Healthy volunteers are not accepted for the study and have a non-probability sampling approach. Through the formation of a strong biological database, this project hopes to facilitate the development of cancer research and enhance therapy approaches in precision medicine.

The “Safety and Immunogenicity Study of the New dHER2 Vaccine to Treat HER2-positive Metastatic Breast Cancer” (NCT00140738), conducted by GlaxoSmithKline, is an open-label Phase I/II trial with the aim of determining the immunogenicity and safety of the dHER2 vaccine among female patients who are at least 18 years of age with advanced HER2-positive metastatic breast cancer. The research involves two arms: first-line therapy (Group A) and second-line therapy (Group B), wherein the participants can get a maximum of 18 vaccine doses for three cycles. Inclusion demands HER2 overexpression or amplification of the gene, having good organ function, and metastatic disease of particular organs; exclusion involves past chemotherapy (in the case of first-line participants), severe cardiovascular conditions, autoimmune illnesses, being pregnant, or receiving immunosuppressive treatment. The trial tracks patients for a year after treatment, with a view to assessing the vaccine’s safety, immune response, and capacity to retard the development of disease.

Kyushu University sponsors the “Clinical Trial of GAIA-102 for Refractory/Relapse Neuroblastomas and Other Malignant Pediatric Solid Tumors” (NCT05608148), a Phase 1 intervention trial to test the safety and efficacy of GAIA-102 in children and young adults (ages 1-24) with resistant neuroblastoma or malignant solid tumors with pulmonary metastasis. The research involves three cohorts: Cohort A, where GAIA-102 is given separately to ascertain its safety and optimal dose for Phase II; Cohort B, where GAIA-102 is used together with Dinutuximab, Filgrastim, and Teceleukin to treat neuroblastoma; and Cohort C, where GAIA-102 is used in combination with Nivolumab. Eligible patients for the study should have failed two or more standard treatment regimens, have a performance status of 50 or higher, and no brain metastases or active autoimmune diseases. The trial, initiated in October 2022 and anticipated to finish in August 2027, assesses primary endpoints like dose-limiting toxicity at the completion of Cycle 1 (28–56 days) and secondary endpoints, including objective response rate, disease control rate, progression-free survival, overall survival, and frequency of adverse events over two years.

The “Carbon-11 Butanol: Whole Body Radiochemical and Radiation Safety” trial (NCT04050800), funded by Weill Medical College of Cornell University, was an initial Phase 1, open-label imaging study intended to evaluate the radiochemical and radiation safety of Carbon-11 butanol in healthy volunteers by PET/CT imaging. Carried out between September 2020 and January 2021 with five participants enrolled, the study entailed intravenous infusion of 555 MBq of Carbon-11 butanol and subsequent serial PET/CT scans every three minutes for two hours to measure whole-body biokinetics quantitatively. Safety measures encompassed vital signs, electrocardiograms (ECGs), and clinical laboratory tests prior to and post-administration, with radiation exposure calculated based on the MIRD formalism. Subjects had to be healthy adults between 18 and 89 years of age, with normal hemodynamic function, no serious medical conditions, and no recent history of radiation, drugs, or vaccines. The main outcomes were radiation safety by time-activity curve analysis of internal organs and clinical safety parameters like vital sign changes, ECG intervals, and renal and hepatic function markers. Secondary outcomes were time-activity curves and cumulative distribution volumes in cerebral regions, lending themselves to calculations of repeatability coefficients in future research endeavors.

The “Clinical Utility of Pediatric Whole Exome Sequencing” trial (NCT03525431), funded by the University of California, San Francisco, was an open-label, interventional diagnostic trial that sought to assess the clinical utility of whole exome sequencing (WES) in undiagnosed pediatric cases. Carried out between August 2017 and May 2022 with 529 enrolled patients, the research examined the performance of WES in the diagnosis of genetic diseases like intellectual disability, seizures, congenital malformations, metabolic disorders, and neurodegenerative disorders. Pediatric patients aged up to 25 years, as long as they had been under follow-up by the UCSF pediatrics department since prior to age 18, were eligible, with at least one biological parent necessary for the collection of specimens. Subjects received WES, variant analysis, and clinical evaluations 6-12 months after results to assess diagnostic yield and impact on patient care. The investigation also assessed variant interpretation challenges, ethical issues, insurance coverage, and access to healthcare across different populations. The main outcome was the number of pediatric patients with a positive exome sequencing result, as defined by the identification of a pathogenic or likely pathogenic variant that accounted for the child’s condition, thus adding to the increasing recognition of WES as a diagnostic tool in pediatric and prenatal clinical practice.

### Challenges and future perspectives

*Scale-up and manufacturing limitations*

Significant obstacles stand in the way of developing novel oral biologic drug delivery systems from research to large-scale manufacturing. Large-scale manufacturing is challenging due to the intricacy of nanocarrier compositions, including their composition, stability, and reproducibility. The final product’s consistency is affected by variables such drug loading efficiency, surface modification, and particle size variability, necessitating strict quality control procedures (58). The cost and complexity of manufacturing are further increased by specialized machinery and sophisticated processing methods including spray drying, high-pressure homogenization, and microfluidics. Since proteins and peptides are extremely susceptible to temperature, humidity, and mechanical stress, another challenge is ensuring the long-term stability of biologics within nanocarriers during storage and transportation. The creation of scalable, economical production techniques that preserve the integrity and operation of the nanocarrier systems is necessary to get over these constraints. These issues could be resolved by developments in AI-driven process optimization, automation, and continuous manufacturing, which would lower production costs and increase batch-to-batch consistency (59).

*Regulatory considerations for oral biologic delivery*

Oral biologic nanocarrier regulatory approval is beset with challenges due to manufacturing, safety, and efficacy concerns. Biologics are required to be subjected to rigorous testing to ensure stability, bioavailability, and immunogenicity, as opposed to conventional small-molecule drugs. Regulatory agencies such as the FDA and EMA require extensive preclinical and clinical studies to assess the pharmacokinetics, toxicity, and long-term safety of drug formulations involving nanocarriers (60). Possible immunological responses, off-targeting, and the need for reliable biomarkers to determine drug absorption and treatment outcomes are some of the key challenges. Regulatory approval of oral biologics through nanocarriers is also affected by the lack of standard measures. Researchers, regulatory agencies, and pharmaceutical companies need to collaborate to overcome these challenges and establish accurate standards for measuring treatments in terms of nanocarriers. In vitro-in vivo correlation (IVIVC) models and sound analytical techniques will be very important in predicting clinical performance and accelerating the approval process (6).

### Future perspectives

The future of oral biologic delivery relies on the integration of advanced nanocarrier technologies, medication formulation through AI, and individualized medicine strategies. Bioavailability and site-specific delivery will still be improved by advancements such as mucoadhesive, pH-sensitive, and stimuli-responsive nanocarriers (61). Furthermore, intriguing alternatives are provided by cutting-edge technologies such as exosome-mediated transport, 3D printing customized drug formulations, and smart polymers with on-demand drug release capabilities (62). In order to bring these technologies to market, regulatory developments such as expedited approval processes and standardized worldwide criteria will be essential. Oral biologic medication delivery has the potential to transform chronic illness treatment options by increasing therapeutic efficacy and patient compliance with sustained investment in research and development (63).

**Table 1:** Commonly used absorption enhancers and their mechanisms of action

Category	Examples	Mechanism of action
Bile salts	Sodium Deoxycholate, Sodium Taurocholate, Sodium Glycodeoxycholate, Sodium Taurodihydrofusidate, Sodium Glycodihydrofudisate	Form reverse micelles and disrupt membrane, open up tight junctions, enzyme inhibition and mucolytic activity
Chelators	EDTA, Citric acid, Salicylates	Interferes with calcium ions, chelation disrupts intracellular junctions and decreases transepithelial electrical resistance
Surfactants	Sodium Lauryl Sulfate, Laureth-9, Sodium Dodecylsulfate, Sodium Taurodihydrofusidate, Poly Oxyethylene Ethers	Perturbation of intercellular lipids, lipid order, orientation and fluidity Inhibition of efflux mechanisms
Fatty acids and Derivatives	Oleic Acid, Linoleic Acid, Caprylic Acid, Capric Acid, Acyl Carnitines, Mono and Di-Glycerides	Increase fluidity of phospholipid membranes, contraction of actin myofilaments, opening of tight junctions
Cationic Polymers	Chitosan and its derivatives	Combined effect of mucoadhesion and opening of tight junctions via ionic interactions with the cell membrane
Anionic Polymers	Carbopol and polyacrylic acid derivatives	Combined effect of enzyme inhibition and opening of tight junctions through removal of extracellular calcium ions
Acylcarnitines	Lauroyl-l-Carnitine Chloride, Palmitoylcarnitine Chloride	Membrane disruption, Opening of tight junctions with a calcium independent mechanism

## Conclusion

Oral delivery of biologics, including proteins and peptides, remains a major pharmaceutical challenge, despite considerable advances. New approaches, such as nanocarriers, enzyme inhibitors, mucoadhesive formulations, and PEGylation, have been demonstrated to improve bioavailability and therapeutic efficacy. Further improvements in the area of drug absorption and site-dependent release have been made through targeted delivery systems using pH-sensitive nanocarriers. Such new therapies, such as orally available GLP-1 receptor agonists and monoclonal antibodies, provide real growth for oral biologic drugs. Problems of large-scale manufacturing, stability, and regulatory approval, however, remain. The way forward is to optimally train drug formulation techniques toward improving cost-effectiveness and clinical translation for universal patient access.

## Declaration

The authors declare that Artificial Intelligence (AI) tools, including language models, were utilized solely for grammatical correction, sentence rephrasing, and language refinement during manuscript preparation. All scientific content, concepts, data analysis, and interpretations were generated and validated entirely by the authors without reliance on AI for content creation.
